# Optimized modelling and application of exhaust gas recirculation performance evaluation of turbocharged diesel engine

**DOI:** 10.1098/rsos.172112

**Published:** 2018-06-27

**Authors:** Chuan-lei Yang, Xiang-huan Zu, He-Chun Wang, Yin-yan Wang

**Affiliations:** College of Power and Energy Engineering, Harbin Engineering University, Harbin 150001, People's Republic of China

**Keywords:** turbocharged diesel engine, exhaust gas recirculation, performance evaluation, optimal decision-making, optimization platform

## Abstract

Aimed at the problem of exhaust gas recirculation (EGR) performance evaluation and optimal EGR rate determination of turbocharged diesel engines, an optimized decision-making method, based on grey theory and entropy weight, was proposed. The internal combustion pressure, fuel consumption rate, NO_X_, CO and smoke were selected as the decision-making targets and the initial decision-making model was established based on the traditional grey decision-making theory. According to the characteristics and optimization requirements of EGR, the optimal compromise between combustion and emission performance is proposed to transform into decision-making target weighting problem, then an optimized subjective weighting method based on expert scoring and grey relational analysis is proposed. Finally, the entropy weight method was used to solve the objective weight and the optimized multi-objective grey decision-making model was established, which can not only weaken the human error of subjective empowerment, but also fully explore the intrinsic relationship of the evaluation indexes. At last, an optimization simulation platform for EGR performance evaluation based on MATLB/GUIDE was designed and established. The results show that the optimization simulation platform can effectively improve the efficiency of simulation calculation, which is more convenient for practical engineering applications. The optimized method can successfully realize EGR performance evaluation and optimal EGR rate determination under different working conditions. The decision-making result was consistent with the present EGR control strategies, which provide a new research idea for EGR performance optimization.

## 1. Introduction

Exhaust gas recirculation (EGR) is the main measure to reduce NO_X_ emissions of diesel engines. The main process is to introduce a part of the exhaust gas into the intake pipe, mixed with fresh air and enter the cylinder to re-enter the combustion process [1,2]. The key to EGR technology is to ensure enough exhaust gas be introduced into the intake pipe and determine a best EGR rate [3]. The basic requirement of EGR is reducing NO_X_ emissions as much as possible while having a minimal impact on particles and other pollutant emissions.

At present, most EGR research works are focused on experimental performance research [4,5], EGR control [6] and so on. Owing to different operating characteristics of EGR under different working conditions, it is difficult to evaluate EGR performance through the present conventional assessment methods. The method commonly used is obtaining the performance parameters of a diesel engine under different operating conditions and different EGR rates through many tests. Based on the test data, different researchers establish various evaluation criteria by subjective judgement to achieve the best EGR rate and make a whole-working-condition optimal EGR rate MAP. The main purpose of different researchers is to ensure good power and economic performance of diesel engines and achieve good emission performance at the same time. However, the criteria vary depending on the aim of the experiment and subjective judgement. For example, Yang [5] proposes the criterion that the particle emission of 13-working-point does not exceed the principle of the original machine when determining the best EGR rate; through many calculations and analyses by BOOST model, Zheng [7] proposes to choose the best EGR rate without overturning the torque, increasing the fuel consumption rate and the soot at first, then consider the emission performance of 13-working-point, and ultimately, the optimal EGR rate can be achieved. Zhang [8] proposes taking the particulate matter as not exceeding the original machine as the basic principle; then taking into account the degree of increase in fuel consumption, improvement of NO_X_ emission and other comprehensive factors, high EGR rate should be selected at low-load conditions and small EGR rate should be selected at high-load conditions. Other researchers, such as Du [9], are using a similar approach. Although an optimal EGR rate can be obtained by each approach, there are some common shortcomings. These methods mainly rely on data sizes and experts’ subjective understanding. Although it can give full play to the professional expertise, it is too subjective and lacks clear theoretical support and guidance. In addition, due to the limitations of the test conditions, sometimes we cannot get enough experimental data, it will increase the difficulty of decision makers' comprehensive judgements and the existing method will be not applicable.

In present EGR research works, there are few relevant studies about the EGR performance evaluation and the optimal EGR rate determination. The EGR performance evaluation and optimal EGR rate determination can be considered as typical multi-objective decision-making problems. Therefore, the multi-objective grey decision-making method is used, which has unique advantages in the decision-making problems of selecting the best scheme for a number of programmes [10] and widely used in aerospace, electronic and other fields [11–17]. Owing to the shortcomings of subjective weighting in the traditional model, more and more optimization models are proposed to improve the reliability of the decision-making results [18–21]. However, different optimization methods are limited to a specific range.

The optimal EGR rate problems involve the trade-off between diesel engine combustion and emission performance under different conditions. Regardless of the traditional decision-making methods or the optimization methods proposed, it cannot satisfy the characteristics and requirement of EGR optimization. Therefore, this paper proposes to transform the optimal compromise between combustion and emission performance into mathematical problems, the trade-off problems are replaced by decision-making targets’ weighting problem and an optimized weighting method is finally proposed based on expert scoring, grey correlation analysis and entropy weight. In addition, to avoid the inconvenience caused by the programming process, but also to improve the efficiency of modelling and simulation calculation, an optimization simulation platform for EGR performance evaluation based on MATLB/GUIDE is designed and established.

In contrast with the existing evaluation methods, the proposed method in this paper is applicable to both large data and poor data occasions. The optimization model is integrated with the EGR optimization characteristics under different working conditions; it can not only achieve the same purpose as the existing method, but also satisfies subjectivity and objectivity. The modelling and simulation efficiency can be improved effectively by the optimization simulation platform which is more convenient for engineering applications. In addition, it extends the engineering application of multi-objective grey decision making, and also provides a new idea for EGR performance optimization of turbocharged diesel engines.

## 2. Basic model of grey theory

### 2.1. Multi-objective grey decision-making model

*Step 1:* Construct the corresponding set of situations according to the event set and the strategy set. Assume that *A* = {*a*_1_, *a*_2_ … *a_n_*} is the event set, the strategy set is *B* = {*b*_1_, *b*_2_ … *b_m_*}, the situation set is *s* = {*s_ij_* = (*a_i_*, *b_j_*)|*a_i_* ∈ *A*, *b_j_* ∈ *B*} and $u_{ij}^{\left(k\right)}(i=1,2\cdots ,n;j=1,2,\cdots m)$ is the effect sample value of the situation under the target.

*Step 2*: Choose the targets and each target needs to determine its effectiveness measure:

2.1 $$r_{ij}^{\left(k\right)}=\frac{u_{ij}^{\left(k\right)}}{\underset{i}{\max}\underset{j}{\max}\{u_{ij}^{\left(k\right)}\}}$$

called the upper effect measure, which is mainly used to measure the degree of the albino value that deviates from the maximum whitening value;

2.2 $$r_{ij}^{\left(k\right)}=\frac{\underset{i}{\min}\underset{j}{\min}\{u_{ij}^{\left(k\right)}\}}{u_{ij}^{\left(k\right)}}$$

called the lower effect measure, which is mainly for the degree of the albino value deviation from the lower limit;

2.3 $$r_{ij}^{\left(k\right)}=\frac{u_{i_{0}j_{0}}^{\left(k\right)}}{u_{i_{0}j_{0}}^{\left(k\right)}+|u_{ij}^{\left(k\right)}-u_{i_{0}j_{0}}^{\left(k\right)}|}$$

called the medium effect measure, where $u_{i_{0}j_{0}}^{\left(k\right)}$ is the moderate effect of the specified effect under the target.

*Step 3:* Solve the same effect measure matrix of situation set according to the effect measure of each target:

2.4 $$R^{\left(k\right)}=(r_{ij}^{\left(k\right)})=\left[\begin{array}{cccc} r_{11}^{\left(k\right)} & r_{12}^{\left(k\right)} & \cdots & r_{1m}^{\left(k\right)}\\ r_{21}^{\left(k\right)} & r_{22}^{\left(k\right)} & \cdots & r_{2m}^{\left(k\right)}\\ \cdots & \cdots & \cdots & \cdots \\ r_{n1}^{\left(k\right)} & r_{n2}^{\left(k\right)} & \cdots & r_{nm}^{\left(k\right)} \end{array}\right].$$

*Step 4:* Establish the decision weight *η_k_*(*k* = 1, 2, … *s*), where $\sum_{k=1}^{v}\eta_{k}=1$ and solve integrated effect measure *r_ij_* and integrated effect measure matrix of situation *s_ij_*

2.5 $$r_{ij}=\sum_{k=1}^{s}\eta_{k}∙r_{ij}^{\left(k\right)}$$

and

2.6 $$R=(r_{ij}^{})=\left[\begin{array}{cccc} r_{11}^{} & r_{12}^{} & \cdots & r_{1m}^{}\\ r_{21}^{} & r_{22}^{} & \cdots & r_{2m}^{}\\ \cdots & \cdots & \cdots & \cdots \\ r_{n1}^{} & r_{n2}^{} & \cdots & r_{nm}^{} \end{array}\right].$$

*Step 5:* If $\underset{1\leq j\leq m}{\max\{r_{ij}\}}=r_{ij_{0}}$, then $b_{j_{0}}$is the optimal strategy to event *a_i_*; if $\underset{1\leq j\leq m}{\max\{r_{ij}\}}=r_{ij_{0}}$, then $a_{i_{0}}$ is the optimal event to strategy *b_j_*; if $\underset{1\leq j\leq m}{\max\{r_{ij}\}}=r_{i_{0}j_{0}}$, then $s_{i_{0}j_{0}}$is the optimal situation.

### 2.2 Grey correlation analysis model

The grey relational analysis theory is an important branch of the grey system theory [19,22–24]. The basic steps are as follows.

Step 1: the original sequence: *X*_0_(*t*) = {*x*_0_(1), *x*_0_(2), … , *x*_0_(*n*)} specifies the reference data sequence, also called the parent sequence. *X_i_*(*t*) = {*x_i_*(1), *x_i_*(2), … , *x_i_*(*n*)} is the sequence of data to be compared, also known as the subsequence.

Step 2: make *ξ_i_*(*k*) the correlation coefficient for sequence *X*_0_(*t*) and *X_i_*(*t*) at time *k*:

2.7 $$\xi_{i}(k)=\frac{\underset{i}{\min}\underset{k}{\min}|x_{0}(k)-x_{i}(k)|+0.5\underset{i}{\max}\underset{k}{\max}|x_{0}(k)-x_{i}(k)|}{\left|x_{0}(k)-x_{i}(k)|+0.5\underset{i}{\max}\underset{k}{\max}|x_{0}(k)-x_{i}(k)\right|},$$

where 0.5 is the resolution factor, usually between 0 and 1.

Step 3: Calculate the average of the correlation coefficients at each time of sequence *X_i_*(*t*), i.e. the degree of correlation of the subsequence *X_i_*(*t*) to the parent sequence *X*_0_(*t*):

2.8 $$r_{i}=\frac{1}{N}\sum_{k=1}^{N}\xi_{i}(k).$$

## 3. Algorithm of optimization decision-making and exhaust gas recirculation performance evaluation

### 3.1. Evaluation index selection

The effect of different EGR rates on the diesel engine combustion and emissions is different and how to get a reasonable compromise is the key to evaluate EGR performance. Both combustion and emission performance of the diesel engine should be taken into account when choosing the evaluation indexes. Therefore, the fuel consumption rate, in-cylinder explosion pressure, NO_X_, smoke and CO are selected as the evaluation targets, which can reflect the dynamic performance, economic performance and emission performance of the diesel engine comprehensively.

### 3.2. Establishment of decision-making target weight

The key to determine the optimal EGR rate is how to reasonably solve the trade-off between diesel engine combustion and emissions performance. Taking into account the EGR operating features under different operating conditions, the paper proposes to transform the ‘trade-off’ into mathematical problems. As each evaluation index represents different performance of diesel engine and considering the important role that target weights play in the decision-making model, the compromise between diesel engine combustion and emission performance is proposed to be achieved by adjusting the target weight value *η_k_* (*k* = 1, 2, 3, 4, 5), where *k* represents the fuel consumption rate, cylinder burst pressure, NO_X_, smoke and CO, respectively.

The operating characteristics of EGR under different conditions are more complicated and it is obvious that neither the subjective weighting method nor the objective weighting method can satisfy the requirement of the EGR performance evaluation. Therefore, it is reasonable to adopt an integrated weighting method.

#### 3.2.1. Establishment of NO_X_ weight

As is well known, the main purpose of EGR is to reduce NO_X_ emissions and the optimization requirements of EGR vary with operating conditions. Therefore, the weight of NO_X_ should be determined at first and the expert scoring method is proposed. According to the related literature, the basic principles of EGR control of marine diesel engines are as follows.

When diesel engine is under idling and warming conditions, no EGR cycle is used; when diesel engine is under low-speed conditions, a smaller EGR rate is used. When diesel engine is under over-acceleration conditions, the lower EGR rate should be adopted. As the speed increases, the EGR rate should also increase accordingly, but it must be controlled within a certain range. When diesel engine is under high-speed and high-load conditions, a larger EGR rate should be adopted.

Based on the above principles, the five decision-making targets are considered equally important. Therefore, each initial weight is 0.2 and the initial decision-making results can be obtained. Analysing the results and comparing with the actual optimization features of EGR, the value of *η*_3_ can be adjusted by adverse deduction. The values of *η*_3_ are 0.2, 0.25, 0.27, 0.3, 0.31, 0.32, 0.33, 0.34, 0.35, 0.36, 0.37, 0.38, 0.39, 0.40, 0.45, 0.50 in order and the final comprehensive optimization weight varies depending on the value of *η*_3_. The basic rules for *η*_3_ can be obtained as follows.

When the diesel engine is under low-speed conditions, the NO_X_ emission concentration is low and in order to ensure the stability and economy of the diesel engine, it is suitable to take a lower EGR rate, thus making the NO_X_ weight *η*_3_= 0.3. When the diesel engine is under high-speed and high-load conditions, the NO_X_ emission concentration is high and in order to ensure the necessary emissions, it is suitable to adopt a higher EGR rate, thus making NO_X_ weight *η*_3_= 0.5. When the diesel engine is under medium-speed conditions, NO_X_ weight *η*_3_ = 0.4.

#### 3.2.2. Establishment of initial weight of all indexes

After determining the NO_X_ weight, the next step is how to assign the weight of the remaining targets. As each index represents a different performance of diesel engine, there must be a certain relationship between them which cannot be determined by intuitive observation and judgement. Therefore, this paper introduces the grey relational analysis method to calculate the importance of the relationship between other indexes and NO_X_, and the weight of all indexes can be obtained.

NO_X_ is defined as the main index and its values (including the original machine value) under different EGR rates were defined as the parent sequence, the other four evaluation indexes are defined as secondary indexes and their values (including the original machine value) at different EGR rates were defined as the subsequence, the correlation coefficient *r_i_* (*i* = 1, 2, 3, 4) between the other four evaluation indexes and NO_X_ can be obtained. Known as *η*_3_ and *r_i_*, the other four decision-making target weight values *η_k_* (*k* = 1, 2, 4, 5) can be solved by the formula *r_i_*(1 − *η*_3_), and then the initial weight of all indexes is established.

#### 3.2.3. Establishment of comprehensive weight

The establishment of the initial weight mainly depends on the current EGR optimization rules, in which the subjective judgement occupies the main factors, and although it can use the advantage of expert experience, it has high subjectivity. To fully explore the internal relations between different decision targets, this paper adopts the entropy weight method to solve the objective weights and the final comprehensive weight can be achieved at last.

As a typical objective weighting method, the entropy method can evaluate multiple evaluation objects by using multiple indexes. It can effectively reflect the implicit data information and enhance the differences of indexes [25,26]. The greater the difference of index values, the more important the object and the higher the weight value. According to the index changes, the weight value of each index can be calculated, which can provide a reliable basis for the comprehensive evaluation of multiple indexes. The basic steps are as follows.

Step 1: There are *n* evaluation objects and *m* evaluation indexes to be evaluated. The original data matrix can be constructed as follows:

$$X=\left[\begin{array}{cccc} X_{11}^{} & X_{12}^{} & \cdots & X_{1m}^{}\\ X_{21}^{} & X_{22}^{} & \cdots & X_{2m}^{}\\ \cdots & \cdots & \cdots & \cdots \\ X_{n1}^{} & X_{n2}^{} & \cdots & X_{nm}^{} \end{array}\right],$$

where *X_ij_* (*i* = 1, 2, … , *m*; *j* = 1, 2, … *n*) is the value of *j*th evaluation object under the *i*th index.

Step 2: The original matrix is converted into proper dimensionless indexes. Calculate the characteristic proportion of the *j*th evaluation object under the *i*th index:

3.1 $$P_{ij}=V_{ij}/\sum_{i=1}^{m}V_{ij}.$$

Step 3: Calculate the entropy of the *i*th index:

3.2 $$e_{j}=\frac{-1}{\ln(m)}\sum_{i=1}^{m}P_{ij}\cdot \ln p_{ij},$$

where the difference *V_ij_* is higher and *e_j_* lower.

Step 4: Solve the entropy of each index:

3.3 $$W_{j}=\frac{d_{j}}{\sum_{=1}^{m}d_{j}},$$

where *d_j_* is the difference coefficient of the *i*th index; the higher the *d_j_*, the greater the amount of information provided by the index.

The original evaluation matrix *X* is constructed by the corresponding values of the evaluation indexes under different EGR rates. The objective weight *η*_0*k*_ (*k* = 1, 2, 3, 4, 5) is obtained by the entropy weight method, and the comprehensive weight *η*_1*k*_ (*k* = 1, 2, 3, 4, 5) is finally obtained:

3.4 $$\eta_{1k}=\frac{\eta_{k}\eta_{0k}}{\sum_{k=1}^{n}\eta_{k}\eta_{ok}}.$$

The optimization method is based on the EGR control strategy. The grey correlation analysis is used to obtain other evaluation indexes' relevance with NO_X_ and the correlation can be used as the basis for solving the weights of other indexes, which can satisfy the basic requirements for EGR. In addition, the entropy weight method is introduced to weaken the problem caused by the subjective weighting and improve the objective rationality of the evaluation result.

### 3.3. Steps to establish the optimization model

For the EGR performance evaluation and decision making: event set *A* = {*a*_1_}, the event is the best EGR rate decision. Strategy set *B* = {*b*_1_, *b*_2_ · · · *b_m_*} consists of the *m* decision-making programme and *b_m_* represents the different EGR rate. The decision-making evaluation targets are the fuel consumption rate, in-cylinder explosion pressure, NO_X_, smoke and CO and their corresponding weights are *η*_1_, *η*_2_, *η*_3_, *η*_4_ and *η*_5_, respectively. The situation of each EGR rate is carried out under the same experimental conditions, and $u_{ij}^{\left(k\right)}$ represents the measurement value of each decision-making index under different conditions and EGR rates. As the fuel consumption, cylinder burst pressure, NO_X_, CO and soot are the lower the better, the lower effect measure is selected. The specific decision modelling steps are as follows:

Step 1: Establish the effect sample matrix $u_{ij}^{\left(k\right)}$ and solve the same effect measure matrix according to formulae (2.1)–(2.3).

Step 2: According to different working conditions of diesel engine, the initial subjective weight *η_k_* (*k* = 1, 2, 3, 4, 5) is obtained by the subjective weighting and grey relational analysis, then the objective weight *η*_0*k*_ (*k* = 1, 2, 3, 4, 5) is obtained by the entropy weight method and the comprehensive weight *η*_1*k*_ (*k* = 1, 2, 3, 4, 5) is finally obtained.

Step 3: Plug *η*_1*k*_ into formula (2.5) to obtain the corresponding comprehensive effect measure matrix.

Step 4: According to the optimal decision-making principle, the advantages and disadvantages of different EGR schemes are sorted and the optimal EGR rate is obtained.

## 4. Implementation of exhaust gas recirculation performance evaluation optimization platform

**Figure 1.**
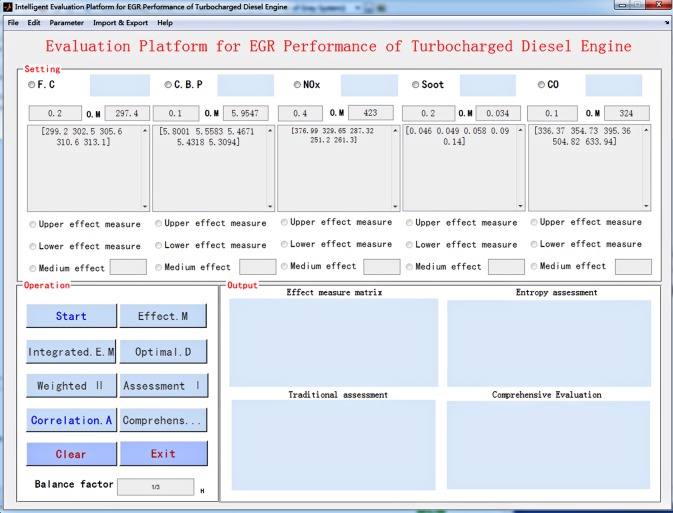
The main interface of the simulation platform.

At present, the implementation of multi-objective decision-making is usually achieved by programming. To avoid the inconvenience caused by the programming process, but also to improve the efficiency of modelling and simulation calculation, the EGR performance evaluation and decision-making GUI is designed and implemented by MATLAB/GUIDE in this paper, and the EGR performance evaluation optimization platform which can work independently is obtained by compiling. The main interface of the simulation platform is shown in figure 1. Users only need to import the EGR parameter data into the optimization platform, the EGR performance evaluation and optimal decision making under different conditions can be completed quickly and the results can be output to the specified location to facilitate post-processing analysis.

## 5. Verification and result analysis

### 5.1 Acquisition of test data

To verify the effectiveness of the optimization method, a certain type of turbocharged diesel engine is regarded as the research object. The main technical parameters of the diesel engine are shown in table 1.

**Table 1.** Main technical parameters of TBD234V12.

project	parameter
power	444 kW (1800 r.p.m.)
cylinderbore × stroke	128 mm × 140 mm
compression ratio	15 : 1
cylinder arrangement	V-shaped 12-cylinder 60° angle
combustion chamber type	direct injection w type

The tests included low, medium and high speeds, where each speed involves 25%, 50% and 75% load, respectively. Part of the operating point test data is shown in table 2, where fc, co, no, soot, cbp represent the fuel consumption, CO, NO_X_, soot and cylinder burst pressure.

**Table 2.** Part of the operating point test data.

OP speed/load	egr/%	fc/g kW^−1 ^h^−1^	co/10^−6^	no/10^−6^	soot/m^−1^	cbp/MPa
900/25%	0	297.4	324	423	0.034	5.9547
	2.4	299.2	336.37	376.99	0.046	5.8001
	4.6	302.5	354.73	329.65	0.049	5.5583
	8.6	305.6	395.36	287.32	0.058	5.4671
	10.4	310.6	504.82	251.2	0.09	5.4318
	11.6	313.1	633.94	231.3	0.14	5.3094
900/50%	0	236.3	309	1093	0.045	7.6462
	2.2	241.6	316.57	1104.5	0.063	7.2545
	4.6	242.7	335.53	1002.6	0.088	7.2108
	7.5	243.9	366.7	943.5	0.084	7.1393
	9.8	246.9	427.84	890.65	0.12	7.0167
	11.5	248.7	503.62	783.6	0.27	6.9568
1500/50	0	215.8	184	1430	0.05	9.2430
	1.8	216.8	164.3	1399.8	0.056	9.0835
	4	217.9	169.9	1365.4	0.064	9.01549
	7.9	220.8	176.9	1240.6	0.078	8.8452
	9.1	224.5	190.4	1119	0.11	8.7263
	11.2	226.1	280.5	1086.4	0.25	8.5216
1500/75	0	200.4	160	2186	0.093	10.8
	1.6	199.8	156.4	2101	0.1	10.5505
	3.9	202.3	164.2	1894	0.13	10.4165
	7.5	205	172.2	1653	0.148	10.2256
	9.7	209.2	206	1521	0.165	10.0584
	11.1	212.2	312.3	1465	0.32	9.8568
1200/50	0	211.2	153	1444	0.075	7.5546
	1.8	211.2	164.3	1436.9	0.09	7.1441
	4.1	214.6	169.9	1408.6	0.103	7.3678
	7.8	216.8	176.9	1343.4	0.148	7.31089
	9.8	222.9	190.4	1221.9	0.184	7.3663
	12.3	224.6	280.5	1113.4	0.42	7.2961
1200/75	0	196.7	149	2012	0.125	9.2060
	2	196.9	156.4	1929.8	0.13	8.8482
	4.1	198.3	164.2	1853.8	0.152	8.6109
	7.9	203.1	172.2	1711.6	0.207	8.5634
	9.7	203.8	206	1534.6	0.28	8.4236
	11.6	208.9	312.3	1399.7	0.51	8.1596

### 5.2. Results analysis

#### 5.2.1. Low-speed conditions

OP1 and OP2 represent the low-speed conditions at 25% load and 50% load, respectively. Taking OP1 as an example, the EGR rates were 2.4%, 4.6%, 8.6%, 10.4% and 11.6%. The effect sample matrix $u_{ij}^{\left(k\right)}$is established as follows:

$$(u_{ij}^{\left(5\right)})=\left[\begin{array}{ccccc} 299.20 & 302.50 & 305.60 & 310.60 & 313.10\\ 5.8001 & 5.5583 & 5.4671 & 5.4318 & 5.3094\\ 376.99 & 329.65 & 287.32 & 251.20 & 261.30\\ 0.0460 & 0.0490 & 0.0580 & 0.0900 & 0.1400\\ 336.37 & 354.73 & 395.36 & 504.82 & 633.94 \end{array}\right].$$

Among them, the row vector represents the fuel consumption rate, CO, NO_X_, soot and in-cylinder burst pressure, the column vector *j* represents the different EGR rates, i.e. the first column means that the EGR rate is 2.4%.

Solve the same effect measure matrix:

$$(r_{ij}^{\left(5\right)})=\left[\begin{array}{ccccc} 1.0000 & 0.9891 & 0.9791 & 0.9633 & 0.9556\\ 0.9154 & 0.9552 & 0.9712 & 0.9775 & 1.0000\\ 0.6663 & 0.7620 & 0.8743 & 1.0000 & 0.9613\\ 1.0000 & 0.9388 & 0.7931 & 0.5111 & 0.3286\\ 1.0000 & 0.9482 & 0.8508 & 0.6663 & 0.5306 \end{array}\right].$$

Solve the initial subjective weight. As OP1 belongs to the low operating point, *η*_3_ = 0.3.

Determine the grey association sequence:

Mother sequence:

$$X0=(\begin{array}{cccccc} 423 & 376.99 & 329.65 & 287.3 & 51.2 & 261.3 \end{array})$$

and subsequence:

$$\begin{array}{rl} X1 & =(\begin{array}{cccccc} 297.4 & 299.2 & 302.5 & 305.6 & 310.6 & 313.1 \end{array}),\\ X2 & =(\begin{array}{cccccc} 5.9547 & 5.8001 & 5.5583 & 5.4671 & 5.4318 & 5.3094 \end{array}),\\ X3 & =(\begin{array}{cccccc} 0.034 & 0.046 & 0.049 & 0.058 & 0.09 & 0.14 \end{array}),\\ X4 & =(\begin{array}{cccccc} 324 & 336.37 & 354.73 & 395.36 & 504.82 & 633.94 \end{array}). \end{array}$$

The correlation coefficients between other evaluation indexes and the NO_X_ index are as follows:

$$r_{i}=(\begin{array}{cccc} 0.2678 & 0.2937 & 0.2091 & 0.2294 \end{array}).$$

Solve the initial subjective weight:

$$\eta_{k}=(\begin{array}{ccccc} 0.1874 & 0.2056 & 0.3000 & 0.1463 & 0.1606 \end{array}).$$

Solve the comprehensive weight *η*_0*k*_ and the integrated weight *η_k_*:

$$\eta_{0k}=(\begin{array}{ccccc} 0.2547 & 0.1805 & 0.1988 & 0.1773 & 0.1887 \end{array})$$

$$\eta_{k}=(\begin{array}{ccccc} 0.2378 & 0.1849 & 0.2971 & 0.1293 & 0.1510 \end{array}).$$

Finally, the comprehensive effect measure matrix can be solved:

$$R=[\begin{array}{ccccc} 0.8852 & 0.9027 & 0.9001 & 0.8735 & 0.8203 \end{array}].$$

In the same way, the comprehensive effect measure matrix with different weights can be obtained, as shown in table 3.

It can be seen from table 3 that when *η*_3_ ≤ 0.3, the comprehensive evaluation value corresponding to low EGR rate is low, and the comprehensive evaluation value obtained by high EGR rate is the lowest, which means that high EGR rate should not be adopted under low-speed conditions. When 0.3 ≤ *η*_3_ ≤ 0.4, the comprehensive evaluation value of high EGR rate is increased. When ≥ 0.36, the comprehensive evaluation value of the high EGR rate is higher than that of lower EGR rate, which shows that a higher EGR rate should be adopted. However, this is inconsistent with the EGR characteristics of marine diesel engines. Particularly, when *η*_3_ ≥ 0.4, the comprehensive evaluation value of high EGR rate is obviously higher than that of low EGR rate. Obviously, it is unreasonable. Therefore, when the diesel engine is in low-speed conditions, the value of *η*_3_ is 0.3.

**Table 3.** Optimization results of different weight.

*η*_3_	*η*_1*k*_	*R*
0.25	[0.2501 0.1954 0.2583 0.1366 0.1596]	[0.8836 0.8948 0.8867 0.8459 0.8223]
0.27	[0.2432 0.1900 0.2787 0.1329 0.1552]	[0.8762 0.8895 0.8844 0.8480 0.8272]
**0.3**	**[0.2378 0.1849 0.2971 0.1293 0.1510]**	**[0.8852 0.9027 0.9001 0.8735 0.8203]**
0.31	[0.2295 0.1793 0.3194 0.1254 0.1464]	[0.8614 0.8789 0.8800 0.8521 0.8369]
0.32	[0.2261 0.1766 0.3296 0.1235 0.1442]	[0.8577 0.8763 0.8788 0.8531 0.8394]
0.33	[0.2227 0.1739 0.3398 0.1216 0.1420]	[0.8540 0.8736 0.8777 0.8541 0.8418]
0.34	[0.2192 0.1713 0.3499 0.1197 0.1399]	[0.8503 0.8710 0.8766 0.8552 0.8442]
0.35	[0.2158 0.1686 0.3600 0.1179 0.1377]	[0.8466 0.8683 0.8755 0.8562 0.8466]
0.36	[0.2124 0.1659 0.3702 0.1160 0.1355]	[0.8429 0.8657 0.8744 0.8572 0.8491]
0.37	[0.2090 0.1633 0.3803 0.1141 0.1333]	[0.8392 0.8631 0.8733 0.8582 0.8515]
0.38	[0.2056 0.1606 0.3904 0.1123 0.1312]	[0.8356 0.8604 0.8721 0.8592 0.8539]
0.39	[0.2022 0.1579 0.4005 0.1104 0.1290]	[0.8319 0.8578 0.8710 0.8603 0.8563]
0.40	[0.1988 0.1553 0.4106 0.1086 0.1268]	[0.8282 0.8552 0.8699 0.8613 0.8588]
0.45	[0.1818 0.1420 0.4609 0.0993 0.1160]	[0.8099 0.8421 0.8644 0.8664 0.8708]
0.50	[0.1649 0.1288 0.5109 0.0901 0.1052]	[0.7916 0.8290 0.8589 0.8714 0.8828]

It can also be seen from the results that the performance ranking of different EGR rates under OP1 conditions is 4.6%, 8.6%, 2.4%, 10.4%, 11.6% and the optimal EGR rate is 4.6%. When the EGR rate is less than approximately 10%, there is only a slight difference between the comprehensive performance evaluation value of different EGR rates. When the EGR rate increased to 10%, the comprehensive performance evaluation value decreased significantly with the increase of EGR rate, which indicates that a higher EGR rate will have significant adverse effect on the overall performance of the diesel engine. Therefore, a smaller EGR rate should be adopted.

Similarly, the comprehensive effect measure matrix of OP2 conditions can be obtained as follows:

$$R=[\begin{array}{ccccc} 0.9040 & 0.8875 & 0.8963 & 0.8719 & 0.8615 \end{array}].$$

It is shown that the optimal EGR rate is 2.2% and when the EGR rate is less than 10%, the difference of corresponding comprehensive performance evaluation value is close. With the increase of EGR rate, evaluation value decreases. In particular, once the EGR rate exceeds 10%, the evaluation value decreases significantly when the EGR rate increases.

The results of OP1 and OP2 show that a smaller EGR rate should be adopted when the diesel engine is at the low-speed, low-load conditions. Analysis of the reason is that the NO_X_ emissions of diesel engine are low and in order to ensure sufficient dynamic performance and economic performance, it is appropriate to use a lower EGR rate, which is also consistent with the current requirements of practical engineering applications.

#### 5.2.2. High-speed conditions

OP3 and OP4 represent the high-speed conditions at 50% load and 75% load, respectively. Make *η*_3_ = 0.5 and the comprehensive effect measure matrix of OP3 and OP4 can be calculated:

$$\begin{array}{rl} & \text{OP}3:\;[\begin{array}{ccccc} 0.8634 & 0.8640 & 0.8970 & 0.9317 & 0.9123 \end{array}]\\ & \text{OP}4:\;[\begin{array}{ccccc} 0.8371 & 0.8515 & 0.8971 & 0.9159 & 0.8871 \end{array}]. \end{array}$$

The performance ranking of the different EGR rates under OP3 and OP4 can be obtained as follows:

$$\begin{array}{rl} & \text{OP}3:\;11.2\%,\;9.1\%,\;7.9\%,\;4\%,\;1.8\%\\ & \text{OP}4:\;9.7\%,\;7.5\%,\;11.1\%,\;3.9\%,\;1.6\%. \end{array}$$

It can be seen from the results that the optimal EGR rates of OP3 and OP4 are 11.2% and 9.7%, respectively. With the increase of EGR rate, the comprehensive evaluation value of each EGR rate increases and the change is more obvious when the EGR rate is greater than 8%. However, when the EGR rate increases to about 11%, the comprehensive evaluation value is reduced.

Analysing the reason for when diesel engine is operating at high-speed conditions, the NO_X_ emissions are relatively high and lower EGR rate has been unable to meet the requirements of reducing NO emissions effectively. Therefore, in order to ensure diesel engine emission performance, the EGR rate should be increased. As a result, the higher EGR rates achieve higher evaluation value. However, for the EGR rate, it is not a case of the higher the better; especially when the diesel engine works in high-speed, high-load conditions, excessive EGR gas will have negative impact on the dynamic performance of the diesel engine, which results in a decrease in the comprehensive evaluation value.

#### 5.2.3. Medium-speed conditions

OP5 and OP6 represent the medium-speed conditions at 50% load and 75% load, respectively. Make *η*_3_ = 0.4 and the comprehensive effect measure matrix of OP5 and OP6 can be calculated as follows:

$$\begin{array}{rl} & \text{OP}5:\;[\begin{array}{ccccc} 0.8850 & 0.8860 & 0.8826 & 0.9007 & 0.9033 \end{array}]\\ & \text{OP}6:\;[\begin{array}{ccccc} 0.8510 & 0.8501 & 0.8525 & 0.8705 & 0.8795 \end{array}]. \end{array}$$

The performance ranking of the different EGR rate at OP5 and OP6 conditions can be obtained as follows:

$$\begin{array}{rl} & \text{OP}5:\;12.3\%>9.8\%>4.1\%>1.8\%>7.8\%\\ & \text{OP}6:\;11.6\%>9.7\%>7.9\%>2\%>4.1\%. \end{array}$$

The results for OP5 and OP6 indicate that the comprehensive evaluation value of each EGR rate increases when the EGR rates increase, and the higher EGR rate is more effective for improving the overall performance of diesel engine when the diesel engine is working at medium-speed conditions. Analysis of the reason is that when the diesel engine is working at medium-speed conditions, NO_X_ emission of the diesel engine gradually increases with the increase of the speed, and it is necessary to increase the EGR rate to reduce the NO_X_ more effectively. Therefore, a higher EGR rate should be used.

In summary, the assessment results demonstrate that when the turbocharged diesel engine is working under low-speed conditions, a smaller EGR rate should be used to balance the power and economy performance of the diesel engine. When the diesel engine is working under high-load conditions, the NO_X_ emission concentration is high and the higher EGR rate should be adopted to ensure the emission performance. However, excessive EGR rates have a negative impact on diesel engines when working under high-speed conditions. This conclusion is consistent with the characteristics of EGR performance of current turbocharged diesel engine, which demonstrates the effectiveness of the optimization method.

In this paper, the EGR rate is limited to less than 15% and the data obtained are limited. However, the authors believe that this limitation does not affect the core of this method because this method is concerned with the EGR performance evaluation and decision making with poor data and uncertainty, which is also the characteristic of this method that distinguishes it from the existing evaluation methods. Therefore, this method can provide some guiding significance for the EGR optimization research of turbocharged diesel engines.

## 6. Conclusion

An improved multi-objective grey decision-making method based on subjective and objective comprehensive weighting is proposed to solve the problem of EGR performance evaluation and optimal EGR rate for turbocharged diesel engines. The best compromise between diesel engine combustion and emission performance is transformed into weighting problem between evaluation targets and the weight optimization method is proposed based on the grey relational analysis and entropy theory, which can meet the operating characteristics of the turbocharged diesel engine and make the decision-making results more reasonable.

The results show that when diesel engine is under the low-speed conditions, the comprehensive performance evaluation value decreases with the increase of EGR rate, the decrease being significant when the EGR rate is greater than 10%. Thus, a smaller EGR rate should be used. When diesel engine is under the high-speed conditions, the comprehensive evaluation value increases with the increase of EGR rate and the change is more obvious when the EGR rate is greater than 8%. However, when the EGR rate increases to about 11%, the comprehensive evaluation value is reduced, thus, a higher EGR rate should be used while not too high. When diesel engine is under medium-speed conditions, the comprehensive evaluation value of each EGR rate increases with the increase of EGR rates, and a higher EGR rate is more effective for improving the overall performance of the diesel engine.

The decision-making results obtained by this method are consistent with the characteristics of the EGR as well as the current best EGR rate control strategies. Therefore, the proposed method can be successfully applied to determine the optimal EGR rate of turbocharged diesel engines under different conditions and the optimization simulation platform can effectively improve the efficiency of simulation calculation.

## Data accessibility

Our data are deposited at Dryad: http://dx.doi.org/10.5061/dryad.gk661 [27].
